# Identifying determinants of care for tailoring implementation in chronic diseases: an evaluation of different methods

**DOI:** 10.1186/s13012-014-0102-3

**Published:** 2014-08-12

**Authors:** Jane Krause, Jan Van Lieshout, Rien Klomp, Elke Huntink, Eivind Aakhus, Signe Flottorp, Cornelia Jaeger, Jost Steinhaeuser, Maciek Godycki-Cwirko, Anna Kowalczyk, Shona Agarwal, Michel Wensing, Richard Baker

**Affiliations:** Department of Health Sciences, University of Leicester, 22-28 Princess Rd West, Leicester, LE2 1TP UK; Scientific Institute for Quality of Healthcare, Radboud University Nijmegen Medical Centre, Nijmegen, Netherlands; Department for Old Age Psychiatry, Innlandet Hospital Trust, N-2312 Ottestad, Norway; Norwegian Knowledge Centre for the Health Services, Oslo, Norway; Department of Public Health and Primary Healthcare, University of Bergen, Bergen, Norway; Department of General Practice and Health Services Research, University of Heidelberg, Voßstraße 2, Geb. 37, 69115 Heidelberg, Germany; Centre for Family and Community Medicine, Medical University of Lodz, Lodz, Poland

**Keywords:** Chronic disease, Guideline adherence, Quality assurance, Healthcare

## Abstract

**Background:**

The tailoring of implementation interventions includes the identification of the determinants of, or barriers to, healthcare practice. Different methods for identifying determinants have been used in implementation projects, but which methods are most appropriate to use is unknown.

**Methods:**

The study was undertaken in five European countries, recommendations for a different chronic condition being addressed in each country: Germany (polypharmacy in multimorbid patients); the Netherlands (cardiovascular risk management); Norway (depression in the elderly); Poland (chronic obstructive pulmonary disease—COPD); and the United Kingdom (UK) (obesity). Using samples of professionals and patients in each country, three methods were compared directly: brainstorming amongst health professionals, interviews of health professionals, and interviews of patients. The additional value of discussion structured through reference to a checklist of determinants in addition to brainstorming, and determinants identified by open questions in a questionnaire survey, were investigated separately. The questionnaire, which included closed questions derived from a checklist of determinants, was administered to samples of health professionals in each country. Determinants were classified according to whether it was likely that they would inform the design of an implementation intervention (defined as plausibly important determinants).

**Results:**

A total of 601 determinants judged to be plausibly important were identified. An additional 609 determinants were judged to be unlikely to inform an implementation intervention, and were classified as not plausibly important. Brainstorming identified 194 of the plausibly important determinants, health professional interviews 152, patient interviews 63, and open questions 48. Structured group discussion identified 144 plausibly important determinants in addition to those already identified by brainstorming.

**Conclusions:**

Systematic methods can lead to the identification of large numbers of determinants. Tailoring will usually include a process to decide, from all the determinants that are identified, those to be addressed by implementation interventions. There is no best buy of methods to identify determinants, and a combination should be used, depending on the topic and setting. Brainstorming is a simple, low cost method that could be relevant to many tailored implementation projects.

**Electronic supplementary material:**

The online version of this article (doi:10.1186/s13012-014-0102-3) contains supplementary material, which is available to authorized users.

## Background

Tailoring implementation interventions to account for the determinants of practice is a common feature of models or frameworks for getting evidence into practice [1,2]. In this paper, we define tailored implementation as implementation interventions to improve professional practice that are planned taking account of prospectively identified determinants of healthcare practice. Determinants are factors that obstruct or enable changes in targeted professional behaviours or healthcare delivery processes. These factors have been referred to as barriers and enablers [3], barriers and facilitators [4,5], or problems and incentives [6]. For example, in an initiative to implement guidelines for antihypertensive and cholesterol-lowering drugs for primary prevention of cardiovascular disease, structured reflection, a questionnaire for physicians, and pilot testing were used to identify determinants, after which a multifaceted intervention was designed, accounting for the determinants [7]. In a Cochrane systematic review of 26 randomised trials of this approach, we found that interventions tailored to prospectively identified determinants are more likely to improve professional practice than no intervention or dissemination of guidelines. However, the methods used to identify determinants and tailor interventions to address them were judged to be only poorly developed [8].

Chronic conditions are increasingly common amongst the ageing populations of many countries worldwide, such conditions including amongst others diabetes [9], dementia [10], and overweight and obesity [10]. The quality of care of chronic conditions is variable at best, and therefore effective approaches are needed for improving care to minimise the burden of exacerbations and complications that individuals will have to cope with and health systems provide care for [11]. If our understanding of the methods of tailored implementation can be improved, the approach has potential to help health systems manage the growing burden of chronic conditions.

Theories of human behaviour [12] or models of practice change [13] may be used to inform the identification of determinants and provide frameworks for categorising them. In a review of frameworks for classifying determinants of practice, some of which used behavioural theories in their development [14], we identified the following broad categories: guideline factors, health professional factors, patient factors, professional interactions, incentives and resources, capacity for organisational change, and social, political, and legal factors [15].

However, although a variety of methods has been used to identify determinants of practice, little research has been undertaken on their validity or feasibility for use in routine initiatives to improve the quality of care [3,15,16].

Methods currently used to identify determinants include: brainstorming, focus groups, analysis of performance data, observations, interviews, and simple or complex questionnaires [16,17]. These methods may be used with various groups, including managers, healthcare professionals, patients or combinations of these, and based in different settings including primary, secondary, and community healthcare. The methods may be used individually or in combinations, and may focus on the subjective perceptions of patients or professionals, or may include more objective methods such as observation [18]. In order to decide on which method, or combination of methods, should be used under different circumstances, evaluation of the methods is required. In particular, it is important to understand how many important determinants are identified by each method.

This study sought to address this lack of evidence by evaluating five different methods for identifying determinants of practice. The aim was to investigate the extent to which the methods identified important determinants and assess their feasibility in use. In particular, we first aimed to compare the extent to which brainstorming, health professional and patient interviews led to the identification of determinants judged to be important, and secondly to determine the additional value of structured group discussions and open questions in surveys of health professionals in identifying further determinants. We also investigated the role of closed questions, derived from the checklist [15] in a questionnaire to samples of health professionals, in identifying the extent to which selected determinants were commonly reported. The study was part of the Tailored Implementation in Chronic Disease (TICD) programme of research that is seeking to advance the methods used in tailoring [2].

## Methods

### Study design

The study took place in five countries, each country team addressing a different chronic condition, as follows: UK (obesity), Germany (polypharmacy in multimorbid patients), Norway depression in the elderly), Netherlands (cardiovascular risk management), and Poland (COPD). The countries were selected because the researchers who developed this EU funded programme of research were based in them; there was no other rationale for the selection of countries. The research team in each country selected the condition to be addressed in their country on the basis of the importance of the condition as they perceived it, and the existence in their country of practice recommendations or guidelines (see Additional file 1 for information on the recommendations targeted in each country). Researchers in each of the five participating countries followed the same protocol.

**Figure 1:**
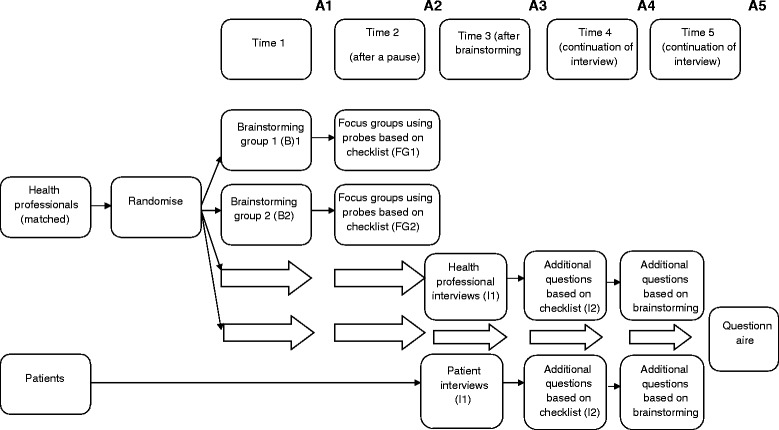
**Schematic protocol, comparative evaluations.**

The study was an evaluation of five methods of identifying determinants (brainstorming, interviews of health professionals, interviews of patients, structured group discussions with health professionals, and questionnaires for health professionals), in which a direct comparison of three methods (brainstorming, health professional interviews, patient interviews) was undertaken, followed by evaluation of the additional value of structured group discussion when undertaken following brainstorming, and the additional value of questionnaires whose design was informed by the brainstorming and health professional and patient interviews, and by reference to the checklist (see Figure 1) [15]. The study received ethics approval from the relevant authority in each country (by the NRES Committee North West - Greater Manchester West for the UK). In order to establish the feasibility of using the various methods, in each country, the research team maintained a diary to record the amount of time spent conducting each of the methods as well as possible difficulties, concerns and benefits that were encountered. In addition, interviews were conducted with a single representative from each of the participating countries. The interview was conducted by one of the researcher team (JK or SA), and sought information on difficulties or challenges in applying the methods, any deviations from the recommended procedures for the methods, and the time taken to conduct and analyse the results of the methods.

### Study population

The study was based in a research centre in each participating country, and took place in either primary or secondary care or both, depending on the particular condition and recommendations being addressed in each country. Samples of healthcare and public health professionals and patients were invited to take part. The samples included health professionals targeted by the clinical recommendations (obesity—general practitioners, practice nurses, dieticians; COPD—general practitioners, practice nurses, pulmonologists; depression in the elderly—physicians or nurses from primary care and psychiatrists and specialist nurses from specialist healthcare; polypharmacy in multimorbidity—GPs and healthcare assistants; cardiovascular risk management—GPs and practice staff). Health professionals were defined as professionals involved in patient care in the targeted clinical domain. Some participants may have had other roles, such as team leaders or clinical teachers, and could also be clinicians or managers. We aimed to include healthcare and public health professionals typical of the population that would be targeted by an intervention to improve adherence to guidelines for the selected condition in each country. In order to identify determinants experienced by a wide range of professionals, we sought to ensure that study populations included a mix of male and female participants with a range of work experience, both in duration and with a mix of clinicians and managers. A recently appointed doctor may have different determinants of practice from a doctor who has been in practice for many years, and managers may have a different perspective on the determinants compared to clinicians.

The patients currently had, or had previously had, the chronic condition of interest. We aimed to include patients at different stages of the condition, different ages, gender and social status. Both health professional and patient participants were provided with a description of the clinical recommendations to be implemented and data on current performance before participating in one of the study groups.

### Methods for identification of determinants

We identified nine commonly used methods for investigating determinants of practice in a literature review, the methods being: brainstorming by the implementation team, analysis of performance data, focus groups (healthcare professionals), focus groups (patients), observations of practice, interviews with healthcare professionals, interviews with patients, simple questionnaires and more detailed questionnaires [15]. The review was undertaken as part of the TICD programme, in parallel with the review of frameworks and typologies for classifying determinants used in developing the checklist [15]. We searched Medline, CINAHL, and PsychInfo for English language articles reporting investigations of determinants of practice; studies involving all types of health professionals and all types of clinical conditions were included. In the searches, we used terms such as barrier, obstacle, enabler, facilitator, classification, taxonomy, ontology, theory, and framework. The search strategy is reported with the report of the checklist [15].

An online, two round, Delphi procedure was used to reach a consensus amongst the investigators from all five countries on which of these methods should be evaluated in our study. The research team of each country was asked to identify five respondents to complete a questionnaire. The respondents included both researchers interested in methods of implementation and clinical professionals with interest in the chronic conditions addressed in our study. Patients or healthcare managers were not included. Respondents were asked to use a nine-point response format to indicate the extent to which they believed each method for identifying determinants possessed the following six attributes (1 = not at all; 9 = completely); the attributes were feasible, comprehensive, valid, consistent, had reasonable costs, and were relevant. These questions were developed in a face-to-face meeting attended by the research collaborators of all five countries. The responses were entered into a database and the numbers of respondents in each response category tabulated, this information being fed back to participants in the second round. The findings of the second round were presented to a face to face meeting of the research collaborators, at which we reached consensus on including the following four methods: structured group discussions with health professionals, health professional interviews, patient interviews, and health professional questionnaires. These methods were most consistently rated by the respondents as having attributes likely to make them useful and feasible in identifying determinants of practice. In addition, brainstorming was used as a low cost, low intensity method.

### Evaluation of methods

Each country used all five methods to identify the determinants of practice for the chronic condition they were addressing.

1. Brainstorming with health professionals (two sessions with between 6 – 10 participants per country),
2. Structured group discussions after brainstorming with health professionals (two sessions with between 6 – 10 participants per country)
3. Interviews of health professionals (a minimum of 8 participants per country)
4. Interviews with patients (a minimum of 8 patients per country)
5. Questionnaire survey of health professionals based on the checklist derived from previous work within the TICD team (120 participants per country) [15].

Three methods were compared directly with each other (brainstorming, interviews of health professionals, interviews of patients). We also investigated the additional value if any of undertaking structured group discussions following brainstorming, and the additional value of a questionnaire for health professionals designed following the completion of the other four methods, and devised in the light of the issues raised by these methods and with reference to the checklist previously developed in the TICD programme [15]. This design did not enable us to compare all five methods with each other, although it allowed us to contain the numbers of participants that would be required and mirrored the approach commonly used in studies of determinants in which combinations of methods are employed, for example the use of questionnaires to supplement structured reflection and review of other studies in the study referred to above as an example of investigation of determinants as part of tailoring implementation [7].

**Figure 2:**
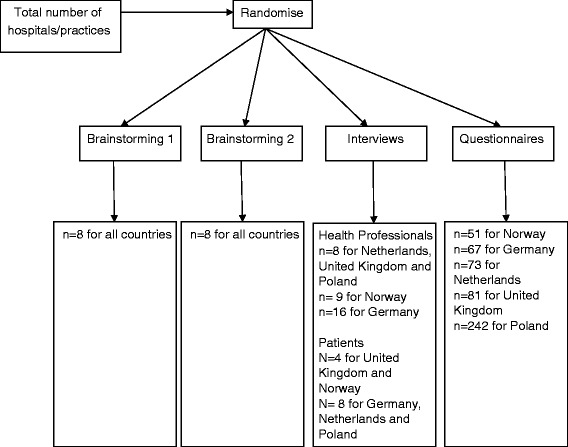
**Randomisation of health professionals.** The target numbers of participants are indicated for each method.

Health professionals were matched and randomly allocated into one of three groups (see above for numbers in each group): a group session comprised of an initial brainstorming phase followed by a structured group discussion; interviews with health professionals; questionnaire (Figure 2). If, after the randomisation, health professionals did not wish to participate in the brainstorming session or interviews then they were asked to complete the questionnaire. With the exception of the brainstorming/structured group discussion groups, no participant completed more than one method. Patients who agreed to participate were assigned to a group for interviews of patients. A schematic representation is shown in Figure 1. The sample sizes were chosen on largely pragmatic grounds, to enable both diversity of participants and the numbers that would typically be manageable in an implementation project. Participants were recruited through letters or emails sent to eligible individuals or practices. For example, in the UK, emailed invitations to take part were sent to general practices interested in research in the east midlands region of the country.

Participants randomised to complete the brainstorming then structured group discussion initially completed a brainstorming session, and after a short break the group discussion drew on the checklist as a prompt [15] to structure the discussion. Interviews with health professionals and patients were either conducted face to face or by telephone. The interviews were semi-structured in approach; a single interview guide was used by each country to produce an interview schedule appropriate for the topic concerned, the checklist being used for additional prompts during the interviews. All interviews were recorded and transcribed.

**Interview guide on which condition specific interview schedules were based in each country**

1. Please can you tell me about your experience of caring for people with condition X (professionals); please can you tell me a little about your experience of having condition X (patients).
2. Care for patients with condition X does not always reflect up to date research evidence about the best way to help patients. This means that patients do not benefit from the best research evidence. We are trying to understand why this might be. Can you tell me, from your experience, what you think sometimes explains this (*i.e.*, what the barriers to evidence-based care are)?
3. Are there any other barriers that you think might be relevant?
4. Which do you think are most important?
5. In your experience, what can help ensure that care does reflect current best evidence?
6. Are there any other enablers that you think might be relevant?
7. Which do you think are most important?
8. Thank you very much for your participation in this study.

The questionnaire was based on the checklist, and was developed using the results of the interviews and brainstorming/structured group discussions. The questionnaire included closed questions with Likert format answers to the five same statements used in all countries for each of their recommendations (although translated into the local language, with a back translation procedure being used to check stability of interpretation):

1. I feel that this recommendation is feasible and practical to undertake in my setting.
2. I feel this recommendation fits with my current practice.
3. I have the knowledge required to implement this recommendation.
4. The benefits of implementing this recommendation outweigh the effort of implementing it.
5. I intend to implement this recommendation.

These items were chosen with reference to the checklist, and the literature undertaken in developing the checklist; we selected checklist domains that appeared commonly in the literature as presenting barriers or enablers to implementation [15]. In addition, country teams included additional questions derived from the checklist that were judged to be relevant to the clinical topic and setting. Respondents to the questionnaire were asked to indicate the extent to which they agreed with the determinants above, using the following five-point scale: fully disagree, disagree, neither agree nor disagree, agree, fully agree. We combined the ‘agree’ and ‘strongly agree’ responses to enable calculation of the proportion of respondents regarding their ability to implement the recommendation favourably. Open questions were also included inviting respondents to highlight any other determinants not covered by the closed questions.

### Measures

The principal measure used to evaluate methods for identifying determinants was the number of plausibly important determinants identified by each method. Plausibly important determinants were defined as ‘a factor for which there was a consensus in the national research teams that it would plausibly inform the design of an intervention’. To inform the design of an intervention, a determinant should firstly have more than a small effect on performance, and secondly, it should be possible to address the determinant in the context of a practical implementation intervention. If a determinant only has a small effect, addressing it in an implementation intervention will not lead to much improvement in care. If addressing a determinant requires an intervention that is not feasible to use, such as the employment of a large number of additional staff or the building of new healthcare facilities, we concluded that we could not plausibly address it. The plausibly important determinants were, therefore, the determinants to concentrate on in tailoring implementation interventions because we expected that it would be possible to deliver interventions to address them and that improved adherence to the recommendations might follow. It should be noted that we did not undertake pilot implementation studies to test our assessments of the importance of individual determinants; furthermore, the research teams in each country may have had different interventions available to them, and an intervention judged not plausible in one country may have been plausible in another. Plausible importance is, therefore, a judgment influenced by context, rather than an absolute property of a determinant. We focus on the plausibly important determinants in this paper (findings on the determinants not judged plausibly important are included in Additional file 2).

To identify the plausibly important determinants from amongst all determinants identified, the following standard procedure was used by the research teams in each country (these teams included a mix of researchers with expertise in health services research and clinical researchers familiar with the clinical field). Each country was asked to rate the determinants using the following criteria, using a five-point scale:

1. How important is the determinant in influencing current practice (as judged by the research team): 1 = very low; 5 = very high (*i.e*., important in determining practice)
2. To what extent can the determinant be addressed: 1 = very difficult; 5 = very easily (*i.e*., it is likely that interventions could be applied to address the determinant).

A single researcher in each country undertook this, with discussion with other researchers within countries, with discussion across countries being used to promote consistency. In the case of disagreements, final decisions were taken by the study co-ordinators (JK, SA, RB). Determinants were classified as plausibly important if they scored at least four for both the above categories. In addition, the total numbers of unique determinants as well as the plausibly important determinants for each method were determined. A unique determinant was defined as a determinant identified by only one method, determinants that were not unique being identified by more than one method. If a method identifies a large number of determinants not identified by any other methods, it may be necessary to include this method as one to be used in investigating determinants. The determinants were also classified by the national research teams according to the checklist developed in earlier work [15].

### Data analysis

The analysis was descriptive only; we did not consider statistical tests appropriate in view of the diversity of the topics and countries. The data were loaded into a database, and we first summarised the extent to which the three initial methods (brainstorming, health professional interviews, and patient interviews) identified plausibly important determinants. We simply enumerated the determinants identified by different methods, in the context of different countries and different chronic conditions. In this analysis, the total numbers of plausibly important determinants were calculated, and the numbers identified by each method alone and those identified by any of the other four methods included in the study. We then investigated the number of additional plausibly important determinants identified by either structured focus groups and or open questions on the questionnaire. We recorded whether determinants were identified only by one method (defined as unique determinants), or by more than one method. We also classified the identified determinants by the domains of the checklist [15], and calculated the mean score in response to the closed questions for the guideline recommendations of each country.

## Results

Seventy-two health professionals (between 10 and 18 in each country) participated in the brainstorming and structured group discussions, 49 health professionals (between 8 and 16 in each country) took part in health professional interviews, 32 patients (4 – 8 per country) took part in the patient interviews, and 514 (67–242) health professionals completed questionnaires. The number of plausibly important determinants identified varied according to country (Table 1). Norway and Germany identified the greatest number of plausibly important determinants (167 and 155 respectively) while Poland identified only 31. Despite Germany identifying a large number of plausibly important determinants, only 11 were classified as unique (*i.e*., identified by only one method), although in the other countries a third or more determinants were classed as unique. The checklist categories to which the determinants related are shown in Table 2. Incentives and resources, and individual health professional factors, were the most common. Relatively few determinants were classified as guideline factors, capacity for organisational change, or social, political, and legal factors. This pattern was generally repeated for all five countries. Table 3 shows the numbers of determinants by domain identified in the interviews of health professionals and patients.

**Table 1:** **Comparison between countries of determinants identified by one method only** (**unique**) **and determinants identified by more than one method**, **in each country**

	**United Kingdom** **(obesity)**	**Norway** **(depression in the elderly)**	**Netherlands** **(cardiovascular risk management)**	**Poland** **(COPD)**	**Germany** **(polypharmacy in multimorbidity patients)**	**Total**
Unique determinants – Not identified by any other method	43 (39.4)	77 (46.1)	62 (44.6)	9 (29.0)	11 (7.1)	202 (33.5)
Identified by at least one other method	66 (60.6)	90 (53.9)	77 (55.4)	22 (71.0)	144 (92.1)	399 (66.5)
Total	109 (100)	167 (100)	139 (100)	31 (100)	155 (100)	601 (100)

N (%).

**Table 2:** **Plausibly important determinants identified by all 5 methods and classified by checklist domain** [15]

**Domain**	**United Kingdom**	**Norway**	**Netherlands**	**Poland**	**Germany**	**Total**
1. Guideline Factors	16	24	8	2	3	53
2. Individual Health Professional Factors	31	51	18	6	36	142
3. Patient Factors	18	36	18	10	15	97
4. Professional Interactions	6	14	28	0	33	81
5. Incentives and Resources	28	30	49	13	41	161
6. Capacity for Organisational Change	4	12	16	0	2	34
7. Social, Political and Legal Factors	6	0	2	0	12	20
8. Miscellaneous	0	0	0	0	13	13

**Table 3:** **The numbers of plausibly important determinants identified by interviews of health professionals or patients**, **by domain**

**Domain**	**Health professionals**	**Patients**
1. Guideline Factors	2	2
2. Individual Health Professional Factors	34	13
3. Patient Factors	13	12
4. Professional Interactions	14	6
5. Incentives and Resources	30	8
6. Capacity for Organisational Change	3	0
7. Social, Political and Legal Factors	4	3
8. Miscellaneous	4	0

### Comparison of brainstorming, health professional interviews and patient interviews

Brainstorming and health professional interviews identified the greatest number of plausibly important determinants, with brainstorming identifying more than three times as many determinants as patient interviews (Table 4). Of the unique determinants, 51.8% were identified by brainstorming, 34.5% by health professional interviews, and 13.7% by patient interviews. In all countries, more than half the determinants were identified by more than one method, although more than one third were classed as unique in Norway, the Netherlands and the UK.

**Table 4:** **A comparison of three methods for identifying plausibly important determinants (brainstorming, health professional interviews and patient interviews)**

**Method**	**Number of determinants not identified by any other method** **(unique determinants)**	Number of determinants Identified by at least one other method*	**Total**
Brain Storming amongst health professionals	72 (37.2)	122 (62.8)	194 (100)
Health Professional Interviews	48 (31.6)	104 (68.4)	152 (100)
Patient Interviews	19 (30.2)	44 (69.8)	63 (100)

*other methods include brainstorming, structured focus groups, open questionnaire, patient interviews, professionals’ interviews.

N (%).

### Additional value of the structured focus groups and questionnaire open questions

Both structured group discussions following brainstorming, and, to a lesser extent, open questions in a survey, identified additional plausibly important determinants (Table 5). Both methods contributed unique determinants, although relatively few were identified by the open questions.

**Table 5:** **Additional value of structured focus groups and open questions on questionnaire in identifying plausibly important determinants**

**Method**	**Not identified by any other method**	**Number Identified by at least one other method**	**Total**
Structured Focus Group in addition to Brainstorming	52 (36.1)	92 (63.9)	144 (100)
Open questions in addition to the questionnaire	10 (20.8)	38 (79.2)	48 (100)

N (%).

### Closed questions for each recommendation

Five closed questions were used per recommendation in each country. The mean score for all five questions per country are summarised in Table 6. Respondents indicated that most of the recommendations were implementable, with the exception of recommendation one for the UK and recommendations three and six for Norway.

**Table 6:** **Mean percentage of responses either agree or strongly agree (standard deviation) to the five questions of the closed questionnaire**

**UK**		**Norway**		**Netherlands**		**Poland**		**Germany**	
**Rec.**	**Mean (SD)**	**Rec.**	**Mean (SD)**	**Rec.**	**Mean (SD)**	**Rec.**	**Mean (SD)**	**Rec.**	**Mean (SD)**
Determine degree of overweight	72.4% (+10.2)	Social contact	80.8% (+15.2)	BP control in raised risk	83.0% (+5.8)	Smoking cessation counseling	83.4% (+4.8)	Structured medication counseling	66.6% (+19.7)
Assess willingness to change	71.1% (+6.8)	Collaborative care	59.8% (+17.3)	BP control in cardiovascular disease	87.7% (+3.5)	Grade breathless	86.3% (+5.4)	Use of medication schedules	92.2% (+6.1)
Offer management	78.8% (+4.4)	Depression care manager	51.0% (+28.3)	Cholesterol control in raised risk	78.9% (+6.1)	Information for the patient	87.9% (+5.3)	Avoid inadequate medication	59.4% (+15.2)
Consider referral	53.1% (+24.9)	Counseling	72.2% (+11.5)	Cholesterol control in cardiovascular disease	88.2% (+2.1)	Inhaler use education	91.8% (+4.2)		
		Mild depression	58.4% (+16.4)	Lifestyle advice	81.4% (+2.5)				
		Severe depression, recurrent or chronic depression, dysthymia	47.4% (+21.5)	Assess risk in chronic kidney disease	75.1% (+6.4)				

Rec **=** recommendations.

### Feasibility

#### Recruiting participants

Successful recruitment of healthcare professionals and patients for interviews varied between the participating countries, but was assisted by the presence of networks of practices interested in research, as in Germany and the UK.

In some instances, the recruitment of GPs proved difficult due to their busy workloads, and the absence of financial incentives seemed to further contribute to the difficulty in those countries in which reimbursement for professionals’ time was not available. Moreover, paper based invitations to participate were less effective than electronic communications. The Norwegian team faced difficulties recruiting patients who were able to discuss their illness and how it related to the recommendation, possibly because of cognitive difficulties or because the recommendations or the task were not presented to the patients in an understandable way.

#### Interviews of professionals and patients

Generally positive attitudes were expressed by each of the participating teams towards the use of interviews as they appeared to yield more in-depth findings than that of questionnaires. Some felt that those healthcare professionals who agreed to participate were the most enthused and engaged with the topic area and so provided the most significant feedback. There were significant time costs associated with the transcription and analysis of each of the interviews as well as the time implications with the interviews themselves. The diaries showed that interviews required the most time of all the methods.

#### Brainstorming and structured group discussions

The methods yielded a wide array of issues associated with each of the chronic conditions, and they informed the interview schedule design, which enabled the key topics to be further explored and reinforce the opinions expressed in the group sessions. Some of the participants were familiar with the methodology, and, in the opinion of some research teams, these methods together yielded the most important plausible determinants. However, some felt the initial silent phase in the brainstorming groups was artificial and often informal discussions broke out regardless of protocol. The transcription and analysis of the group sessions took time, but given that each team ran only two group sessions in comparison to several interviews, the time costs were not as large as with the interviews.

#### Questionnaires

Each of the participating countries experienced significant problems with the questionnaire, and arguably out of each of the methods it was regarded as the most problematic. Firstly, there were problems in achieving adequate response rates, exacerbated by the use of paper based questionnaires when necessary instead of electronic questionnaires. The Norwegian team was unable to obtain email addresses from various healthcare professional organisations due to data protection issues, and so was reliant on paper-based questionnaires. The paper based questionnaires together with follow up reminder letters were costly.

## Discussion

### Main findings and interpretation

In this study, we investigated different methods for identifying those determinants of practice that may be addressed in tailored implementation interventions, which we have termed plausibly important determinants. Each of the methods was able to identify such determinants, although brainstorming and interviews with health professionals identified the greatest number of determinants in all countries. The open questions of the questionnaire and interviews with patients identified fewer determinants. Although the number of determinants identified by interviews with patients was relatively low (in comparison to other methods) nearly a third were classified as unique. The findings suggest that there is no single best method for identifying determinants, but that a combination of methods should be considered, chosen depending on the guideline or recommendations being implemented. Thus, although the large number of unique and plausibly important determinants identified by brainstorming suggests that it could be used as a relatively quick and inexpensive method to identify a large number of determinants, if patients or health professionals are particularly affected by the targeted recommendations, interviews of patients and health professionals should be undertaken as well. Therefore, a combination of brainstorming, and health professional and patient interviews may be adequate in the case of many chronic conditions. In view of the effectiveness of the structured group discussions in generating additional determinants, the use of the checklist or similar prompting mechanism is likely to be helpful.

It is striking how many determinants were identified in each country. The numbers per country did vary, from 167 in Norway (depression in the elderly) to 31 in Poland (COPD), but it is not clear whether this variation is accounted for by the conditions addressed, or whether the perceptions of professionals and patients and their propensity to report problems in care differ between countries. The finding does suggest, however, that tailored implementation interventions should not be assumed to be transferrable between conditions or countries.

We used a systematic approach and several different methods, and identified 601 plausibly important determinants in total (a mean of 120 per country). This finding has implications for implementation strategies; if there are so many determinants of practice that should be accounted for, the process of tailoring will potentially be challenging. For example, it would be difficult, if possible at all, to address 120 determinants in any implementation program. An alternative might be addressing determinants at the level of the individual, since the number of determinants relating to an individual health professional is likely to be fewer, but the problem of large numbers of determinants will recur if several individuals are involved. In our study, we eliminated determinants that we judged were unlikely to be important, or not amenable to change through an implementation strategy (see Additional file 2). It is possible that our decisions on some determinants were wrong; the process for selecting the most important determinants to address require developing and testing in future work.

### Strengths and limitations

To our knowledge, this is the first study to compare the effectiveness of different methods of identifying determinants of practice to inform tailoring, in different chronic conditions in different countries. A standard protocol was followed in each country, and we believe the procedures followed in each country were broadly consistent. However, there may have been some variation; for example, randomisation of participants to study groups was undertaken separately in each country without central control, and therefore some inconsistency may have crept in. Likewise, the classification of determinants as plausibly important was undertaken within each participating country, leading to opportunities for some inconsistency.

We are unable to judge whether or not all the determinants have been identified, since there is no gold standard method against which to compare the methods used in this study. It is not possible to determine whether the determinants we have identified are genuinely the most important to address in implementing change, and we cannot be certain that our assessments of the importance of the determinants and the extent to which they are amenable to change are valid. We will, however, assess the effectiveness of the tailored interventions by clustered trials in each country, and explore the validity of the determinants addressed through process evaluations of the trials [19–24] of the plausibly important determinants identified, the majority were classified as individual health professional factors and incentives and resources. Relatively few were classified as capacity for organisational change, and social, political, and legal factors, which would be difficult to address in the context of an implementation intervention [14]. The questionnaire was designed in the light of the findings of the interviews since we could not be blinded to the findings of the interviews. We were unable, therefore, to directly compare the ability of questionnaires to elicit determinants with the other methods.

### Comparison with literature

Despite a high number of studies on barriers for change, we have identified little other research into different methods of identifying determinants. Bosch *et al*. [17] investigated the methods used in 20 quality improvement studies, finding that a variety of methods were used. Most were qualitative methods such as interviews of professionals or patients, and it was not possible to recommend which methods should generally be employed.

### Practice implications

This study has advanced understanding of determinants of practice by showing that many can be identified by making explicit the process by which identified determinants are assessed and those most important to address selected, and by showing that there is no overall ‘best buy’ of method for identifying determinants. Different methods tend to lead to the identification of rather different sets of determinants, and consequently use of a combination of methods is more likely to lead to the identification of the key plausibly important determinants than use of any single method alone. The nature of the guideline recommendations being implemented should be taken into account, as patients or health professionals may have particular views in relation to some recommendations. Our findings suggest that brainstorming with a structured group discussion (using a checklist to prompt suggestions) and one additional method (*e.g*., interviews of health professionals, interviews of patients) should identify a high proportion of determinants in relation to the costs and time involved in conducting each method.

Once the determinants of practice to be targeted have been identified, interventions are required to address them. This step in the process of tailored implementation is not considered in this paper. However, our findings do have implications for the process of tailoring implementation to account for determinants. It is difficult to devise an intervention to address each and every determinant. Tailoring is therefore likely to require a further set of choices to be made about which determinants should be prioritised, or which interventions may be likely to address, at least in part, several determinants. In the TICD research programme, a study is underway to investigate approaches to tailoring [2].

## Conclusions

Tailored implementation is a complex approach, a key step of which is the identification of determinants of practice. This step involves selecting which methods to use and deciding which of the determinants are important to address. A selection of methods is available for identifying determinants, and in most implementation initiatives, a mix of methods should be used in order to identify most of the important determinants. Because a large number of determinants are likely to be identified, a process is required to extract from the many those few that can be practically addressed in implementation interventions, with consequent improved adherence to recommendations. In the absence of such a process, implementation risks remaining an often ‘hit or miss affair,’ with the impact on practice improvement being unpredictable and inadequate. The development and evaluation of systematic approaches to select the most important determinants is now required.

## Additional files

Additional file 1:
**The chronic conditions and clinical practice recommendations addressed in each country.**
Additional file 2:
**The determinants not categorised as plausibly important.**

## Abbreviation

TICD: The Tailored Implementation of Chronic Disease programme of studies
