# Supplementary material for: Identifying determinants of care for tailoring implementation in chronic diseases: an evaluation of different methods
Source: Implement Sci. 2014 Aug 12;9:102. doi: 10.1186/s13012-014-0102-3 (PMC4243773; doi:10.1186/s13012-014-0102-3)
Supplement: Additional file 1: — The chronic conditions and clinical practice recommendations addressed in each country. [file 13012_2014_102_MOESM1_ESM.docx]

**Additional file 1. The chronic conditions and clinical practice recommendations addressed in each country.**

**Germany – Multimorbidity**

1. Structured medication counselling – Every 6 months a review of medications (in patients taking more than 5 medications) is completed. The following topics are discussed; compliance, improvement in symptoms, adverse drug reactions, need for the drug, the side effects of the drugs and the patient’s thoughts and feelings.

2. Consequent use of medication schedules – All patients taking permanent or on demand drugs should have a hand written medication schedule. Medication schedules should be comprehensive, available and contain all essential information.

3. Avoidance of potentially inadequate medication (PIM) - Since 2010, a German list summarises 82 drugs which should be avoided in older patients, as they have a higher risk for adverse drug reactions. The number of PIM prescriptions should be reduced.

Recommendations based on national guideline.[26]

**Norway – Depression in the elderly**

*Target population - people aged 65 years and over with depression*

1. Social contact. Primary care physicians and other health care professionals should discuss social contact with elderly patients with depression, and recommend actions (e.g. group activities) for those who have limited social contact

2. Collaborative care. All municipalities should develop a plan for collaborative care for patients with moderate to severe depression

3. Depression care manager (DCM). Primary care physicians should offer patients with moderate to severe depression regular contact with a depression care manager

4. Counselling. Primary care physicians or qualified health care professionals should offer self-assisted advice to elderly patients with depression

5. Mild depression. Primary care physicians should not prescribe antidepressants to patients with mild depression. They may prescribe antidepressants to such patients if the patient has previously suffered from moderate to severe depression and has responded to antidepressants

6. Severe depression, recurrent depression, chronic depression and dysthymia. Primary care physicians should offer these patients a combination of antidepressant medication and psychotherapy

Recommendations taken from national guidelines.[25]

**United Kingdom – obesity**

*Target patient population – all adults with a diagnosis of overweight or obesity*

1. Determine the degree of overweight or obesity (BMI and waist circumference)

2. Assess lifestyle, co-morbidities, and willingness to change

3. Management: A multi-component intervention should be offered to encourage increased physical activity, improved eating behaviour, and healthy eating. Drugs may be used in certain groups. The intervention should involve long-term follow up by a trained professional and be tailored to the patient’s preferences, initial fitness and lifestyle.

4. Referral: This is appropriate when the cause is uncertain, surgery is being considered, or there are complex co-morbidities.

Recommendations taken from national guidelines.[22]

**Netherlands – Cardiovascular risk management**

*Target population – all adults all patients with an elevated risk with and without established cardiovascular disease.*

1. Strive for a SBP <140 mm Hg in patients with a 10 years risk of cardiovascular mortality or morbidity ≥ 20%

2. Strive for a SBD <140 mm Hg for patients with CVD

3. Strive for a LDL cholesterol <2.5 mmol/l in patients with a 10 years risk of cardiovascular mortality or morbidity ≥ 20%

4. Strive for LDL cholesterol <2.5 mmol/l for patients with CVD

5. Give lifestyle advice for modifiable risk factors

6. Assess a cardiovascular risk profile for every patient with a chronic kidney disease.

Recommendations based on national guideline.[23] [24]

**Poland: COPD**

*Target patient population – all adults with a diagnosis of COPD.*

1. Brief smoking cessation counseling is effective and every tobacco user should be offered such advice at every contact with health care providers.

2. One of the primary symptoms of COPD is breathlessness. The Medical Research Council (MRC) dyspnoea scale should be used to grade the breathlessness according to the level of exertion required to elicit it.

3. Every patient should receive basic information about the disease and about actions and expected effects of used drugs, making him/her an active, conscious participant in long-term treatment. The health care team should be a source of strength for the patient to adjust to the disease, confidence and an optimistic view of the future. When dyspnoea occurs patients should be encouraged to continue with physical exercise, to avoid the consequences of a vicious circle: shortness of breath - a reluctance to exercise - impaired use of oxygen in muscles - increased demand for oxygen - increased effort of breathing - increased shortness of breath

4. Patients must be educated in the correct use of whatever inhalation device is employed.

Recommendations based on key national publications [19] [20] [21]
