# Supplementary material for: Identifying determinants of care for tailoring implementation in chronic diseases: an evaluation of different methods
Source: Implement Sci. 2014 Aug 12;9:102. doi: 10.1186/s13012-014-0102-3 (PMC4243773; doi:10.1186/s13012-014-0102-3)
Supplement: Additional file 2: — The determinants not categorised as plausibly important. [file 13012_2014_102_MOESM2_ESM.docx]

**Additional file 2. Determinants of practice that were not judged to be plausibly important.**

This file should be read in conjunction with the paper **Identifying determinants of care for tailoring implementation in chronic diseases: a comparison of different methods.** In this file, we present information on the determinants that were not classified as plausibly important. The methods are set out in the accompanying paper.

In addition to the 600 determinants that were classified as plausibly important, a further 609 determinants were identified from the five participating countries (Table a1). The categories of the checklist to which determinants were classified are shown in Table a2. Health professional interviews identified the highest number of determinants that were not classed as plausibly important (Table a3). Open questions identified smaller numbers of determinants (Table a4).

**Table a1. Comparison between countries of unique and non-unique determinants not judged to be plausibly important.**

|  | **United Kingdom** | **Norway** | **Netherlands** | **Poland** | **Germany** | **Total** |
| --- | --- | --- | --- | --- | --- | --- |
| Unique determinants – Not identified by any other method | 19 (43.1) | 85 (46.7) | 50 (45.4) | 38 (41.3) | 26 (14.4) | 218 (35.8) |
| Identified by at least one other method | 25 (56.9) | 97 (56.3) | 60 (54.5) | 54 (58.7) | 155 (85.6) | 391 (64.2) |
| Total | 44 (100) | 182 (100) | 110 (100) | 92 (100) | 181 (100) | 609 (100) |

**Table a2.** **Determinants judged to be not plausibly important classified by checklist domain (Flottorp et al, 2013).**

| **Domain** | **United Kingdom (obesity)** | **Norway (depression in the elderly)** | **Netherlands (cardiovascular risk management)** | **Poland (COPD)** | **Germany (polypharmacy in multimorbidity)** | **Total** |
| --- | --- | --- | --- | --- | --- | --- |
| 1. Guideline Factors | 2 | 5 | 3 | 11 | 0 | 21 |
| 1. Individual Health Professional Factors | 3 | 28 | 12 | 9 | 38 | 90 |
| 1. Patient Factors | 7 | 2 | 8 | 9 | 22 | 68 |
| 1. Professional Interactions | 3 | 6 | 4 | 0 | 19 | 32 |
| 1. Incentives and Resources | 3 | 18 | 10 | 8 | 31 | 69 |
| 1. Capacity for Organisational Change | 1 | 13 | 4 | 7 | 14 | 39 |
| 1. Social, Political and Legal Factors | 6 | 4 | 14 | 10 | 8 | 42 |

**Table a3.** **A comparison of three methods for identifying determinants not judged to be plausibly important (brainstorming, health professional interviews and patient interviews)**. **other methods include brainstorming, structured focus groups, open questionnaire, patient interviews, professionals interviews.*

| **Method** | **Number of determinants not identified by any other method** | **Number of determinants Identified by at least one other method*** | **Total** |
| --- | --- | --- | --- |
| Brain Storming | 58 (37.2) | 98 (62.8) | 156 (100) |
| Health Professional Interviews | 88 (38.1) | 143 (61.9) | 231 (100) |
| Patient Interviews | 29 (35.8) | 52 (64.2) | 81 (100) |

**Table a4.** **Additional value of structured focus groups and open questions on questionnaire in identifying determinants not judged to be plausibly important**

| **Method** | **Not identified by any other method** | **Number Identified by at least one other method** | **Total** |
| --- | --- | --- | --- |
| Structured Focus Group in addition to Brainstorming | 34 (34.0) | 66 (66.0) | 100 (100) |
| Open questions in addition to the questionnaire | 9 (21.9) | 32 (78.1) | 41 (100) |
